# Development of a Breadfruit Flour Pasta Product

**DOI:** 10.3390/foods8030110

**Published:** 2019-03-26

**Authors:** Carmen L. Nochera, Diane Ragone

**Affiliations:** 1 Department of Biomedical Sciences, Grand Valley State University, Allendale, MI 49401, USA; 2 Breadfruit Institute, National Tropical Botanical Garden, Kalaheo, HI 96741, USA; ragone@ntbg.org

**Keywords:** ‘Ma’afala’, *Artocarpus altilis*, gluten-free pasta, underutilized crop, value-added product, indigenous crop cultivar

## Abstract

Breadfruit *(Artocarpus altilis*) is grown throughout the tropics. Processing the perishable starchy fruit into flour provides a means to expand the use of the fruit. The flour can be used to develop new value-added products for local use and potential export. The purpose of this investigation was to develop a pasta product using breadfruit flour, test the sensory qualities of the breadfruit pasta product by sensory evaluation, and evaluate the nutritional composition. ‘Ma’afala’, a popular and widely distributed Polynesian cultivar was used for the study. Nutritional labeling shows that the breadfruit pasta product is high in carbohydrates (73.3%/100 g) and low in fat (8.33/100 g). Sensory evaluation indicates that 80.3% of the panelists (*n* = 71) found the pasta acceptable while 18.3% disliked the pasta. The breadfruit pasta product can provide a nutritious, appealing and inexpensive gluten-free food source based on locally available breadfruit in areas of the world where it can be easily grown.

## 1. Introduction

Breadfruit (*Artocarpus altilis* (Parkinson) Fosberg) is cultivated in more than 90 countries [1,2] throughout the tropics, yet is generally considered an underutilized crop. It is a rich source of carbohydrates, fiber, vitamins, minerals and flavonoids [2,3,4,5,6,7,8,9,10], and contains complete protein [11]. It is also gluten free [6,7]. With its great potential to increase food production in a sustainable and regenerative manner, breadfruit could become an important crop to address food insecurity issues in many tropical areas. Since the pioneering work on breadfruit flour by Loos et al. [12], Arcelay and Graham [13], and Nochera and Caldwell [14], numerous studies have focused on developing and evaluating products using locally grown breadfruit flour as a substitute for imported wheat flour [7,15,16,17,18,19,20].

In the past decade, the interest in gluten-free products has accelerated efforts to use breadfruit in value-added products such as chips, fries, dips, baked goods, desserts, and beverages. It has also driven interest in processing breadfruit into flour. Breadfruit flour products will expand and complement existing and potential markets for the fresh or processed fruit [2].

The emerging breadfruit flour industry currently involves researchers, farmers, cooperatives, and entrepreneurs in Hawaii, Samoa and American Samoa, the Caribbean, Central America, and West Africa who are producing small quantities of flour for local use and for export [2]. Regulatory issues regarding the use of breadfruit flour in North America have been addressed, including US Food and Drug Administration (FDA) approving an application for breadfruit flour to be granted “Generally Recognized as Safe” status [21]. 

The main purpose of this investigation was to develop a nutritious pasta product using only breadfruit flour and no additional flours. The flour was made from ‘Ma’afala’ a Polynesian cultivar of breadfruit. ‘Ma’afala’ is a popular and commonly grown cultivar indigenous to Samoa and Tonga and grown in many other Polynesian and Micronesian islands [22]. This cultivar was selected for micropropagation [1,23] and global distribution based on its excellent horticultural and nutritional attributes, fruit quality, seasonality, and yields [1,10,24,25]. In the past decade, through the Breadfruit Institute’s “Global Hunger Initiative”, thousands of ‘Ma’afala’ trees have been introduced to more than 40 countries [26]. The fruit produces high quality flour containing 7.6% protein, which is similar to rice (7.4%), and higher than many tropical staples. ‘Yellow’ and ‘White’, the cultivars typically cultivated outside of the Pacific region, contain 5.3% and 4.1% protein, respectively [7].

As with other non-cereal and non-grain flours, breadfruit flour does not contain gluten. Glutenin and gliadin are the major protein constituents in gluten. This protein network is responsible not only for volume, texture, viscoelasticity, and rheological properties, but also for cohesiveness and binding properties [27,28]. An anticipated challenge considered when undertaking this investigation was selecting appropriate ingredients that would provide the required binding capacity and deliver a cohesive breadfruit pasta product.

Pasta is a popular commercial food product because of its ease of preparation, palatability, versatility, low cost, nutritional value, and long shelf life. Pasta products can be prepared at home or by food service operations, and also provide a practical, portable, and stable storage form. Wheat flour has been extensively used in the production of alimentary pastas such as macaroni, spaghetti, and other noodle forms. Noodles are an important food product throughout the world [29,30].

Pasta products have previously been developed utilizing a composite mixture of breadfruit and wheat flour [29,30,31], or breadfruit and cassava flour [32]. Our study is the first to develop a pasta product using only breadfruit flour and to determine its sensory qualities and nutritional value.

## 2. Materials and Methods

### 2.1. Harvest and Preparation of the Breadfruit Flour

The breadfruit cultivar, ‘Ma’afala’—see [33,34] for fruit attributes—was utilized for the development of the breadfruit pasta. Mature fruit was harvested by hand from trees in McBryde Garden in the National Tropical Botanical Garden, Kalaheo, Kauai, Hawaii. Washed breadfruit was peeled, and the pulp was sectioned and dried at 80 °C for 24 h. Dried pulp was ground in a mill (Waring) to produce flour that passed through an 80 mesh (180 µm) sieve. 

### 2.2. Preparation of the Breadfruit Pasta Product

**Figure A1:**
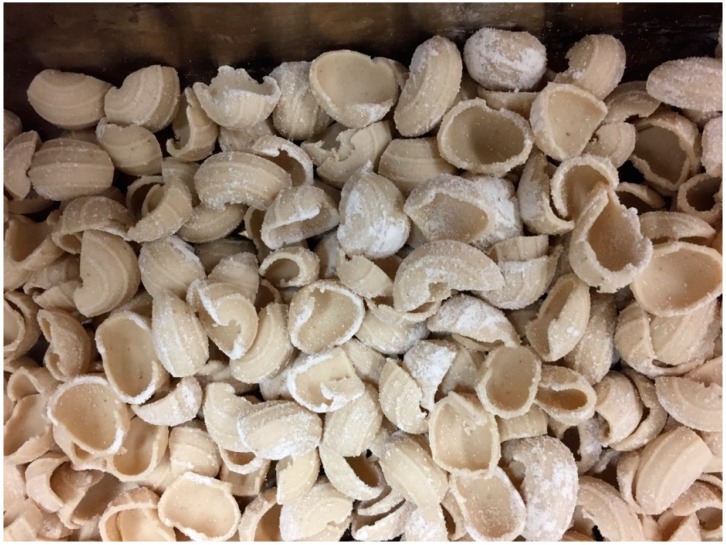
Breadfruit pasta (Orecchiette). Photo© Carmen L. Nochera.

Other than the breadfruit flour, all the ingredients (tapioca starch, salt, psyllium powder, xanthan gum, and coconut oil) were purchased commercially. The dry ingredients were combined in the hopper of a pasta extruder (Arcobaleno AEX 18 pasta extruder). With the machine running slowly, the oil was added followed by the water. The mixture was kneaded for about five minutes resulting in a coarse and crumbly batter. The batter was then extruded using an orecchiette pasta die (Figure A1). The resulting breadfruit pasta was dried in a food dehydrator at 54 °C for about six hours. The recipe formulation is listed in Table 1.

### 2.3. Chemical and Nutritional Analyses of the Breadfruit Pasta Product

Proximate analysis (crude fiber, ash, moisture) was determined for the breadfruit pasta product according to procedures outlined by AOAC, 2005.08 [35]. Nutrition labeling (calories, calories from total fat, total fat, fatty acids (saturated, trans and poly/mono unsaturated fat) cholesterol, sodium, total carbohydrate, dietary fiber, sugars, protein, vitamin D, calcium, and iron) was performed according to procedures outlined by AOAC, 2005.08 [35]. The gluten content analysis of the breadfruit pasta product was performed according to procedures outlined by AOAC, IR061201.2006 [36].

### 2.4. Sensory Evaluation of the Breadfruit Pasta Product

The breadfruit pasta product was tested for acceptability of taste using a hedonic test according to Larmond [37] and Meeilgard [38]. The product was evaluated by 71 untrained panelists. A nine-point verbal category hedonic scale was used: 1, dislike extremely; 5, neither like nor dislike; 9, like extremely. The scale was presented as a line numbered 1–9 with the beginning, middle, and end parameters specified. The pasta was presented without additives. Data obtained from the taste panel were analyzed using the Z test for one proportion. 

The study was approved by the Human Research Review Committee at Grand Valley State University, Allendale, Michigan. Informed consent was obtained from each participant. 

## 3. Results and Discussion

A nutritious breadfruit pasta product was successfully developed. Tapioca starch (*Manihot esculenta*), and fibers such as psyllium (*Plantago ovata*) and xanthan gum (*Xanthomonas campestris*) were incorporated into the breadfruit flour mixture to provide texture, cohesiveness and binding capacity [28,39,40,41,42,43]. Tapioca starch was primarily utilized because of its gluten-free nature, water-holding capacity, and pasting and gelling properties which contribute to texture [40]. Psyllium fiber is usually used as a laxative; however, it can provide strong gelling and binding properties due to its content of arabinose and xylose polysaccharides [42]. Gums and hydrocolloids are mostly polysaccharides. They can also improve texture. Xanthan gum improves the cohesion of starch granules, thereby contributing to the structure of the product [28,39]. Coconut oil was used as it is readily available throughout the tropics. Oils can also contribute to the binding capacity of the mixture [28], and salt and oil contribute to taste.

Corn starch was not used as it can potentially be an allergen [44]. When potato or rice flours were added to the mixture, potato flour produced a dryer, thicker dough, and rice flour resulted in a sticky dough. It was not possible to extrude either mixture into a pasta product.

Results of label analyses based upon proximate analyses are presented in Table 2. Each 2 oz (40 g) serving of breadfruit pasta provided 3.7 g of dietary fiber. Similar results were obtained for a breadfruit bar [16]. There is variability among the reported fiber content of breadfruit [2,10,16]. This may be dependent upon species, maturity, processing, or the type of analysis used for determination of fiber. Ragone and Cavaletto [2] and Turi et al. [10] reported that 100 g of cooked breadfruit can contain up to 7.37 g crude fiber. Fiber has been demonstrated to reduce the incidence of degenerative diseases such as cancer, cardiovascular disease and diabetes [45].

Previous studies have reported that breadfruit is gluten free [6,10]. Analyses of the breadfruit pasta product showed that the pasta contained less than 20 ppm of gluten. According to the FDA, a product must contain less than 20 ppm in order for it to be considered gluten free [46]. Breadfruit offers great potential for use in food product development for those who suffer from celiac disease and gluten allergies. 

**Figure 1:**
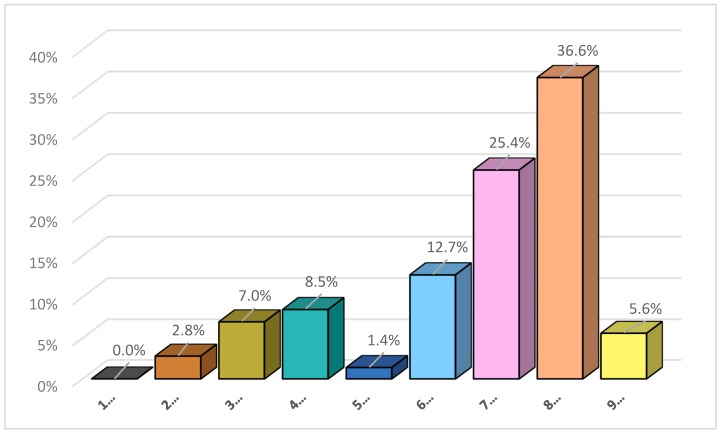
Overall Acceptability of Taste.

Sensory evaluation results are presented in Figure 1. The 9-point scale was collapsed to a 2-point scale: those who responded “Like Slightly, Like Moderately, Like Very Much or Like Extremely” as Group 1 (LIKE), and those who responded “Dislike Slightly, Dislike Moderately, Dislike Very Much or Dislike Extremely” as Group 2 (DO NOT LIKE). The grouping allowed estimation of the proportion of the population who like the breadfruit pasta using the one-sample Z-test. For this sample, 57 of the 71 indicated that they liked the breadfruit pasta. We can report with 95% confidence that the proportion of people who like the breadfruit is somewhere between 71% and 89.5% [0.8028 ± 1.96 × sqrt (0.8028 × (1 − 0.8028)/71) = (0.710, 0.895)]. Since the confidence interval excludes 50%, there is sufficient evidence to conclude that the majority of the tasters liked the breadfruit pasta.

## 4. Conclusions

The major purpose of this investigation was to develop a nutritious, appealing, and inexpensive pasta product based on locally available breadfruit in areas of the world where it can be easily grown, test its sensory qualities, and evaluate its nutritional properties. This research study demonstrated that a breadfruit pasta product can be developed using only breadfruit flour, in this case using flour processed from the fruit of the Polynesian cultivar, ‘Ma’afala’. Sensory analyses showed acceptability, so this breadfruit pasta is a promising value-added product that could potentially compete with other pasta products on the market. 

The glycemic index (GI) reflects the degree to which a food raises the blood glucose [47]. Studies have demonstrated that cooked breadfruit has a low to moderate GI; hence, it can prevent hyperinsulinemia [10,47,48,49]. To date, there have been no published studies on the GI of products developed from breadfruit flour [47]. Therefore, it is recommended that the GI be determined for newly developed breadfruit products. 

The data from this project can help guide efforts in developing new products in which breadfruit flour replaces wheat flour. A recommended first step is to similarly prepare and evaluate breadfruit pasta made from flour processed from other cultivars, such as the widely grown ‘Yellow’ or ‘White’. Diversifying the uses of breadfruit in food product development will continue to enhance its utilization and market potential

## Figures and Tables

**Table 1:** Breadfruit pasta product ingredients.

Ingredients	Grams (g)	Source
Breadfruit Flour	275	McBryde Garden, NTBG, Kauai, Hawaii
Tapioca Starch	178	Harvest Foods, West Michigan
Salt	14	Harvest Foods, West Michigan
Psyllium Powder	9	Harvest Foods, West Michigan
Xanthan Gum	9	Harvest Foods, West Michigan
Water	295	Tap water
Coconut Oil	28.3	Harvest Foods, West Michigan

**Table 2:** Nutritional label analysis.

Analysis	Unit	Result per 100 g	Result per Serving Size 2 oz. Dry (40 g)	Label Declaration	% Daily Value
Calories	-	378	151	150	
Total Fat	g	8.33	3.33	3.5	4
Saturated Fat	g	6.9	2.8	3	14
Trans Fat	g	<0.1	<0.1	0	
Polyunsaturated Fat ^1^	g	0.3	0.1	0	
Monounsaturated Fat ^1^	g	1.2	0.5	0	
Sodium	mg	12	5	0	0
Cholesterol	mg	<1	<1	0	0
Total Carbohydrate	g	73.3	29.3	29	11
Dietary Fiber	g	9.3	3.7	4	13
Sugars	g	1.26	0.5	Less than 1	
Protein	g	2.32	0.93	Less than 1	
Vitamin D	mcg	<0.1	<0.1	0	0
Calcium	mg	86	34	30	2
Iron	mg	1.48	0.59	0.06	4
Potassium	mg	826	330	330	8
Ash ^1^	%	4.58			
Moisture ^1^	%	11.4			

^1^ = Non-mandatory or voluntary label declarations.
